# Effects of pH and MgSO_4_ on Lignin Redeposition and Retention during Brownstock Washing

**DOI:** 10.1021/acsomega.6c06072

**Published:** 2026-10-26

**Authors:** Eashwara Raju Senthilkumar, Maria Sofia Suarez, Fredrik Wernersson Brodin, Tomas Vikström, Gunnar Henriksson, Olena Sevastyanova

**Affiliations:** † Department of Fibre and Polymer Technology, School of Chemistry, Biotechnology and Health, Royal Institute of Technology, Teknikringen 56-58, Stockholm 100 44, Stockholm Country, Sweden; ‡ Södra Cell AB, Södra Innovation and Strategy, Väröbacka 432 86, Halland County, Sweden; § Valmet AB, Gustaf Gidlöfs väg 4, Sundsvall 851 94, Sweden; ∥ Wallenberg Wood Science Centre, School of Chemistry, Biotechnology and Health, Royal Institute of Technology, Teknikringen 56-58, Stockholm 100 44, Stockholm County, Sweden

## Abstract

Process closure in kraft pulp mills requires increased recycling of washing liquors, which can alter liquor composition and promote lignin redeposition during brownstock washing. This study investigates the effect of pH and magnesium ions (Mg^2+^) on lignin retention and removal from pulp fibers in controlled washing experiments. Unbleached and bleached softwood kraft pulps were washed with industrial liquors collected from different stages of the kraft process at a Swedish mill. Lignin redeposition and retention were assessed using kappa number, brightness, and alkaline leaching. Within the alkaline pH range investigated, decreasing pH was generally associated with increased lignin retention on the fibers. NaOH addition improved lignin removal, but the effect plateaued beyond a liquor-dependent threshold. MgSO_4_ addition had little effect at the mill-relevant concentration of 1 g/L, whereas the deliberately elevated concentration of 10 g/L increased lignin retention. Overall, the results demonstrate that liquor chemistry influences the balance between lignin removal and retention during brownstock washing. Because conventional mill-scale indicators such as chemical oxygen demand (COD), dissolved solids carryover, and wash loss were not measured, the results are interpreted specifically in terms of lignin redeposition and retention rather than overall washer performance.

## Graphical abstract

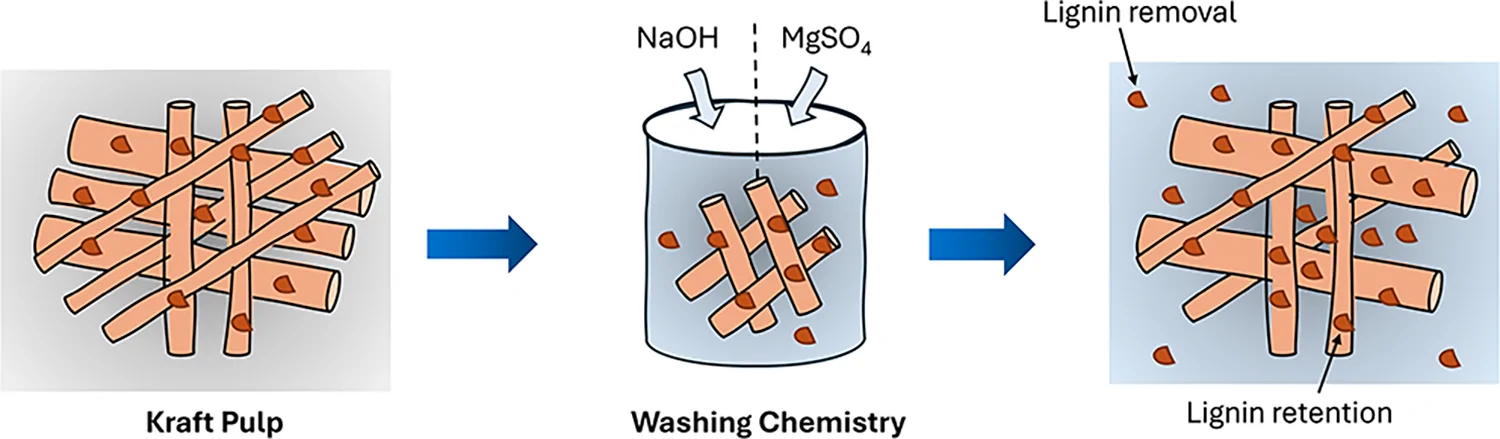

## 1 Introduction

Kraft pulping is the dominant chemical pulping process for producing paper-grade pulps, in which wood chips are digested under strongly alkaline conditions to dissolve lignin and liberate cellulose fibers.^1^ After cooking, brownstock washing (BSW) is required to remove dissolved lignin and residual chemicals before oxygen delignification and bleaching. Washing efficiency has a significant impact on chemical recovery, bleaching costs, environmental performance, and overall mill operations.^2^

Liquor recycling in counter-current washing systems has increased in modern pulp mills as part of efforts to optimize the process and improve environmental sustainability. This reduces freshwater consumption and effluent generation, but can also alter liquor composition and increase the risk of lignin redeposition on pulp fibers.^2,3^ As the degree of system closure increases, nonprocess elements (NPEs), including Ca^2+^ and Mg^2+^, can accumulate in process streams, potentially contributing to scaling, fouling, and reduced washing efficiency.^4,5^ Lignin reattachment can lower pulp brightness, increase the kappa number, and increase the demand for bleaching chemicals.^4,6^ Therefore, understanding how liquor chemistry influences lignin reattachment is important for optimizing brownstock washing.

Lignin redeposition occurs when solubilized lignin fragments and extractives reattach to the surfaces of pulp fibers.^7^ van der Waals forces, hydrophobic interactions, electrostatic attraction, and mass transfer between the fiber wall and the surrounding liquor can all contribute to these processes. The diffusion of dissolved lignin from the fiber wall is also an important factor. Eriksson and Grén^25^ demonstrated that diffusivities differ between the outer and inner fiber-wall layers, with the outer layer being depleted relatively quickly, whereas diffusion from the inner layers is considerably slower. This indicates that residence time and washing temperature are important parameters.

Among the chemical variables, pH and divalent cations can strongly influence lignin behavior in solutions. Under alkaline conditions, kraft lignin behaves as a polyelectrolyte; deprotonation of ionizable groups, including phenolic hydroxyl groups, increases its negative charge and contributes to its solubility and colloidal stability.^8^ As pH decreases, the degree of ionization and the negative charge density of lignin decrease, thereby reducing electrostatic stabilization and favoring lignin association, aggregation, and ultimately precipitation or redeposition.^9,10^ This effect becomes particularly pronounced as the pH approaches the dissociation range of phenolic groups. Because kraft cooking is carried out under strongly alkaline conditions and the alkalinity decreases through subsequent washing operations, changes in lignin ionization can influence its solubility and tendency to reattach to fibers.

Ion-specific effects further complicate brownstock washing. According to the Hofmeister series, different ions influence hydrophobic interactions and colloidal stability to varying degrees.^11^ MgSO_4_ is a kosmotropic salt that can strengthen hydrophobic interactions and affect lignin colloidal stability. At sufficiently high salt concentrations, lignin solubility can decrease, promoting aggregation and precipitation.^12,13^ Early work by Lindström^23,26^ established the colloidal behavior of kraft lignin, showing that both simple and complex metal ions can induce coagulation of lignin sols, with divalent and trivalent cations being particularly effective. Divalent cations such as calcium (Ca^2+^) and magnesium (Mg^2+^) can screen or partially neutralize the negative charges associated with lignin, reducing electrostatic repulsion and thereby favoring aggregation.^3,14^ Calcium ions have also been associated with lignin redeposition and reduced pulp brightness.^3,27^

Magnesium ions play a more complex role, as they can participate in hydroxide formation, facilitate heterocoagulation, and interact with both lignin and cellulose surfaces.^7^ Metal ions such as Mg^2+^ and Ca^2+^ can also interact directly with pulp fibers through ion exchange at carboxyl groups on hemicelluloses and residual lignin.^15–17^ The sorption of Na^+^, Mg^2+^, and Ca^2+^ on kraft pulp fibers has been studied extensively,^18,19^ and the competition between these cations for fiber-binding sites can alter the fiber surface charge and thereby influence lignin adsorption behavior.^20^

The relevance of Mg^2+^ is further increased by the use of magnesium salts in oxygen delignification to protect cellulose, which may result in Mg^2+^ carryover into subsequent process streams. Previous studies have reported magnesium levels corresponding to approximately 1.2 g/L as MgSO_4_ (10 mmol/L Mg^2+^) under mill oxygen-delignification conditions.^3,27^ At such concentrations, pronounced effects on lignin precipitation are not expected. Based on these reported values, approximately 1 g/L MgSO_4_ was therefore selected as the mill-relevant concentration in the present study.

Recent studies have clarified several aspects of lignin redeposition. For example, sodium sulfate (Na_2_SO_4_) has been shown to strengthen hydrophobic interactions, thereby promoting lignin reattachment to pulp fibers during washing.^21^ Temperature also plays an important role: elevated temperatures reduce liquor viscosity and enhance diffusion and mass transfer, but may also influence lignin solubility and aggregation.^1,2,22^ In addition, incomplete black liquor displacement and variations in fiber surface properties can further increase the risks of lignin redeposition.^7^ However, the effects of pH and divalent cations, particularly Mg^2+^, on lignin redeposition remain poorly understood despite their industrial relevance. Previous studies suggest that Mg^2+^ may stabilize lignin dispersions at relatively low concentrations,^3^ whereas higher concentrations have been associated with increased aggregation under alkaline conditions.^7,23^ These observations indicate that the effect of Mg^2+^ may be concentration-dependent and highlight the need for systematic investigations under mill-relevant conditions.

The present study addresses this gap by examining the effects of liquor composition, pH adjustment, and MgSO_4_ addition on lignin redeposition during brownstock washing of unbleached and bleached softwood kraft pulps. By combining controlled laboratory experiments with kappa number, brightness, and alkaline leaching analysis, the study aims to clarify how pH and Mg^2+^ influence the balance between lignin removal and reattachment. The results provide insights into lignin redeposition and identify liquor-chemistry conditions that may help minimize lignin retention and carryover during brownstock washing.

## 2 Materials and Methods

### 2.1 Materials

Kraft pulp and black liquors were obtained from Södra Cell Värö, Sweden, which processes mixed Norway spruce (*Picea abies*) and Scots pine (*Pinus sylvestris*). The samples included both unbleached and bleached pulp as well as liquors from different mill stages: L3 was collected from the bottom of the digester (representing a mixture of weak black liquor and washing liquor from the cooking stage), while L1 and L2 were collected from the inlet and outlet of the pressure diffuser process streams in the washing line. Analytical-grade sodium hydroxide, sulfuric acid, and magnesium sulfate from Sigma-Aldrich were used in the laboratory experiments. All chemicals were dissolved in deionized water. Additionally, three sodium hydroxide solutions were prepared to match the pH of industrial liquors and served as blank references.

### 2.2 Methods

#### 2.2.1 Raw Materials Preparation

Before the experiments, both the pulp and the liquors were pretreated. At the mill, the pulp was washed using twin roll press (TRP) washers. In the laboratory, the pulp was further leached at 2–3% consistency (mass of oven-dry pulp per total suspension mass, expressed as a percentage) in deionized water. The pulp was stirred in deionized water for 30 min to leach residual chemicals. After this 30 min leaching step, 20 L of deionized water was poured over 10 L of pulp suspension in a nylon cloth bag (100 μm mesh) to displace the liquor. The pulp was then centrifuged in an Electrolux 240 centrifuge and dewatered to 30% consistency. The pulp was then resuspended in 20 L of deionized water and centrifuged again with the same conditions as before. The cleaned pulp was stored in a sealed plastic container at 4 °C for no more than 1 week before use.

The liquors were filtered using a Büchner funnel with an internal diameter of 130 mm and grade 1001 Ahlstrom Munksjö filter paper (Finland) to remove fines. The filtered liquors were then stored in plastic bottles. To ensure reproducibility, both raw materials were kept at 4 °C throughout the study and removed immediately before each experiment.

In each trial, 20 g of oven-dry pulp (i.e., 67 g of pulp with 30% dryness) was mixed with 135 mL of corresponding liquor and 3 mL of chemical solution, with water added as needed to achieve a 10% consistency (a slightly higher chemical volume was used in pH-decreasing studies). The liquor and chemical solution were premixed, poured over the pulp, and mixed.

#### 2.2.2 Chemical Preparation

The following procedures were used to prepare the chemical solutions before each trial. Solid NaOH was dissolved in deionized water to match the pH of the corresponding liquors (L1, L2, L3), yielding the blank references B1, B2, and B3. These blanks thus matched the pH of each industrial liquor. Still, they contained no dissolved lignin or process ions, serving as controls to isolate the effect of liquor composition from pH effects.

For pH-increase experiments, three NaOH solutions corresponding to addition levels of 2, 5, and 10 g/L were prepared. Stock solutions (2.3, 5.8, and 11.5 M) were used to obtain the desired concentrations in the final 138 mL mixtures (135 mL liquor + 3 mL chemical solution). As shown in Table 1, for pH-decrease experiments, sulfuric acid (H_2_SO_4_) was added at volumes of 16.8 and 17.6 mL of 1 M H_2_SO_4_ to 135 mL of liquor to achieve the target pH values.

**Table 1:** Preparation of Sulfuric Acid Samples

target pH	volume added (mL)	level of addition (g/l)
12.5	16.8	10.9
12	17.6	11.3

For the MgSO_4_ addition trials, solutions were prepared in deionized water at concentrations of 1, 3, and 10 g/L. Stock solutions with molarities of 0.4, 1.1, and 3.8 M were used to obtain the desired levels in the final 138 mL mixtures (135 mL liquor + 3 mL chemical solution). At the laboratory treatment liquor-to-pulp ratio of 138 mL mixture per 20 g oven-dry pulp (equivalent to 6.9 m^3^/ADT on this experimental basis), these concentrations correspond to nominal MgSO_4_ charges of 6.9, 20.7, and 69.0 kg/ADT, respectively. The 1 g/L condition was selected as the mill-relevant level, whereas 3 and 10 g/L were included as elevated and deliberately supra-industrial conditions to reveal concentration-dependent behavior and support mechanistic interpretation; the 10 g/L result is not presented as a normal operating condition.

## 3 Experimental Section

After sample preparation, the mixtures were transferred into weighed double polyethylene bags, which were then double sealed after as much air as possible was manually removed. The degree of air removal was not quantified; this is acknowledged as a potential source of variability. To simulate industrial conditions, the bags were placed in a Julabo TW20 hot water bath (Julabo GmbH, Seelbach, Germany) at 90 °C for 30 min, preceded by a 5 min acclimation period. As summarized in Table 2, the experimental procedures were grouped into series 1, 2, and 3.

**Table 2:** Experimental Design of the Study^a^ ^,^ ^b^

series	#1: liquors	#2: pH	#3: divalent cations
Pulp	P1, P2	P1, P2	P1
Liquor	L1, L2, L3, B1, B2, B3	L2, L3	L2
Chemical added		NaOH/H_2_SO_4_	MgSO_4_
Level of addition (g/l)		2, 5, 10/10.9, 11.3	1, 3, 10
NaOH and MgSO_4_ Charge (kg/ADT)		13.8, 34.5, 69	6.9, 20.7, 69.0

a MgSO_4_ charges in kg/ADT are based on 138 mL treatment mixture per 20 g oven-dry pulp (6.9 m^3^/ADT).

b P1: Unbleached pulp, P2: Bleached pulp, L1, L2, and L3: Industrial liquors, B1, B2, B3: Blank solutions (without lignin).

### 3.1 Dewatering

The pulp suspension was transferred from the sealed bags onto a 100 μm nylon cloth in a 130 mm Büchner funnel (Figure 1). It was dewatered using vacuum filtration and a hand press to form a pulp cake. Hand pressing was performed by a single operator across all experiments to minimize variability. The filtrate was then passed back through the cake for a second dewatering step, in order to maximize the capture of fine particles and dissolved material that may have passed through on the first filtration. The collected liquor was weighed and stored for subsequent analysis.

**Figure 1:**
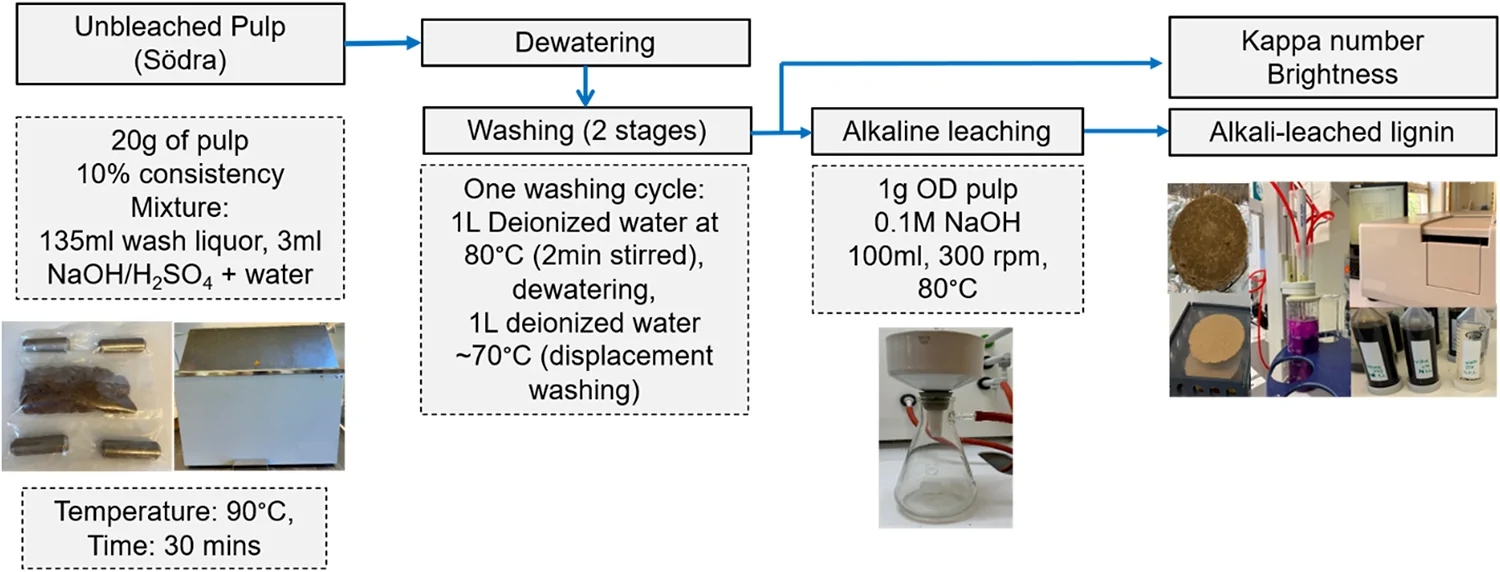
An experimental sketch was followed in this study to perform the washing experiments.

### 3.2 Washing

After dewatering, the pulp was manually stirred in a plastic vessel containing 1 L of preheated deionized water (80 °C) to simulate industrial washing conditions (80–90 °C). Deionized water was used rather than process water to provide a defined baseline free of variable ionic content; it is acknowledged that mills typically use process water or filtrates, which would affect ionic strength and washing behavior. After a 2 min acclimation period, the suspension was transferred to the same Büchner funnel for dewatering. The filtrate was then passed back through the pulp cake and dewatered again. The temperature of the filtrate during recirculation was not measured; it is noted that filtrate cooling during drainage could vary between samples depending on drainage time and liquor composition, potentially affecting lignin solubility. Displacement washing was then performed by pouring 1 L of hot deionized water (∼70 °C) onto the pulp, followed by final dewatering via vacuum filtration. This entire washing cycle—comprising stirring in 1 L hot water, vacuum filtration, filtrate recirculation, displacement with 1 L hot water, and final dewatering was repeated twice, giving two complete washing stages. Two washing stages were selected to approximate typical industrial counter-current washing while maintaining practical experimental throughput. Liquors from each stage were weighed and collected for analysis, and the washed pulp was dried overnight at 40 °C in an air-circulation oven to 30% dryness before further characterization.

### 3.3 Alkaline Leaching

After washing, the pulp was subjected to an alkaline leaching procedure originally developed at STFI and subsequently described by Senthilkumar et al. (2025) to extract loosely bound, redeposited lignin from the pulp fibers. One gram of oven-dry pulp was suspended in 100 mL of 0.1 M NaOH under magnetic stirring at 300 rpm and immersed in an oil bath at 80 °C with an aluminum cover. After 1 min, the mixture was dewatered through a 100 μm nylon cloth in a 74 mm Büchner funnel. The filtrate was passed through the pulp cake a second time, followed by a final displacement step with 50 mL of deionized water at 80 °C. The pulp was collected, and the combined filtrates from leaching and washing were weighed and stored for subsequent analysis.

### 3.4 Pulp Analysis

The pulp’s kappa number was determined according to ISO 302:2015 (E), which specifies the method for measuring residual lignin content. Measurements were performed using an automatic titrator, Mettler Toledo T70. Brightness was measured according to SCAN-CM 11:95. Paper sheets with a grammage of 200 g/m^2^ were prepared using a 110 mm Büchner funnel with Ahlstrom Munksjö grade 1001 filter paper, formed with a PTI 40140 sheet press, and dried overnight at 25 °C and 50–60% relative humidity. Brightness was then measured using a Shimadzu UV-2550 UV–vis spectrophotometer in accordance with ISO 2470, by recording reflectance at 457 nm at four positions on each sheet. The four positions were evenly distributed across the sheet surface to ensure representative coverage. The average value was determined relative to a barium sulfate reference plate.

The liquor obtained by dewatering, washing, and alkaline leaching was quantified by UV–vis spectroscopy using a Shimadzu UV-2550 spectrophotometer. Before measurement, the liquor was filtered and diluted with a pH 10.3 NaOH solution. The absorbance was then measured at 280 nm and normalized to the value at 600 nm. Finally, to determine the lignin content in the solution, Beer–Lambert’s Law was used, with a molar absorptivity coefficient of 24.6 dm^3^ g^–1^ cm^–1^ as follows^24^

1 $$lignin\left(\frac{mg}{gODpulp}\right)=\frac{{(A}_{280nm}-{(A}_{600nm})\times \mathrm{DF}\times V}{\varepsilon \times b\times P}$$

*A*_280nm_: Absorbance value at 280 nm; *A*_600nm_: Absorbance value at 600 nm; DF: Dilution factor; *V*: Volume of filtrate, mL; ε: Mass absorptivity coefficient (L/g·cm); *b*: length, cm; *P*: OD pulp, g.

## 4 Results

### 4.1 Initial Characterization of Pulps and Liquors

Before the experimental procedures, all pulp and liquor samples were characterized, as summarized in Tables 3 and 4. Kappa number and brightness were measured for both pulps. In addition, both pulps were subjected to alkaline leaching to provide values for lignin removal.

**Table 3:** Pulp Samples Characterization

pulp	unbleached (P1)	bleached (P2)
Kappa number	26.3	0.89
Brightness (%)	30.4 ± 0.2	89.63 ± 0.4
Alkali Leached Lignin (mg/g OD pulp)	2.1 ± 0.07	0.61

**Table 4:** Liquor Samples Characterization

liquor	L1	L2	L3
pH	12.4	12.9	13.5
Conductivity (mS/cm)	40.6	48.5 ± 0.1	79.6
Lignin content (g/l)	23.9 ± 1.6	46.1 ± 1.4	101.6
Residual alkali (g/l)	0.2	0.3	10.1

The liquors were analyzed for pH, conductivity, and lignin content, while residual alkali values, determined by acid–base titration, were provided by Södra (Table 4). Liquor L3, collected from the bottom of the digester, represents a different process stream from L1 and L2 and may therefore contain lignin with different molecular-weight distributions and chemical characteristics. However, molecular weight and functional group analyses were not performed in the present study; therefore, such differences cannot be confirmed. L1 and L2 originated from the inlet and outlet, respectively, of the first pressure diffuser, whereas L3 consisted of a mixture of weak black liquor and washing liquor collected from the bottom of the digester.

P1 (unbleached pulp) exhibits low brightness and a high kappa number, consistent with its high residual lignin content. In contrast, P2 (bleached pulp) contains substantially less residual lignin and therefore provides a useful reference for investigating lignin adsorption onto comparatively lignin-free fibers. Among the liquors, L3 has the highest concentration and conductivity, whereas L1 has the lowest values. These differences in pulp and liquor characteristics provide a basis for interpreting the effects of Mg^2+^ addition, pH adjustment, and liquor composition, as discussed in the following sections.

### 4.2 Effects of Liquor Composition on Lignin Redeposition and Removal Efficiency

The impact of liquor composition on lignin retention and removal was evaluated for both unbleached (P1) and bleached pulp (P2) using kappa number, brightness, and alkali-leached lignin measurements (Figure 2). Table 5 compares the results obtained for unbleached pulp after treatment with the industrial liquors and the corresponding blanks. The initial kappa number values were provided by Södra Cell Värö, Sweden. The abbreviations used are as follows: BB = brightness after treatment with blank, LB = leached lignin after treatment with blank, LL = leached lignin after treatment with liquor, and BL = brightness after treatment with liquor. Overall, treatment with the industrial liquors (L1–L3) resulted in greater lignin retention than treatment with the corresponding blanks (B1–B3), as indicated by higher kappa numbers, higher amounts of leachable lignin, and lower brightness (Figure 2a). Among the industrial liquors, those characterized by higher lignin concentrations and conductivity were generally associated with greater lignin retention. The effect of the initial liquor pH was less pronounced, while lignin removal generally increased as the lignin concentration and conductivity of the industrial liquors decreased.

**Figure 2:**
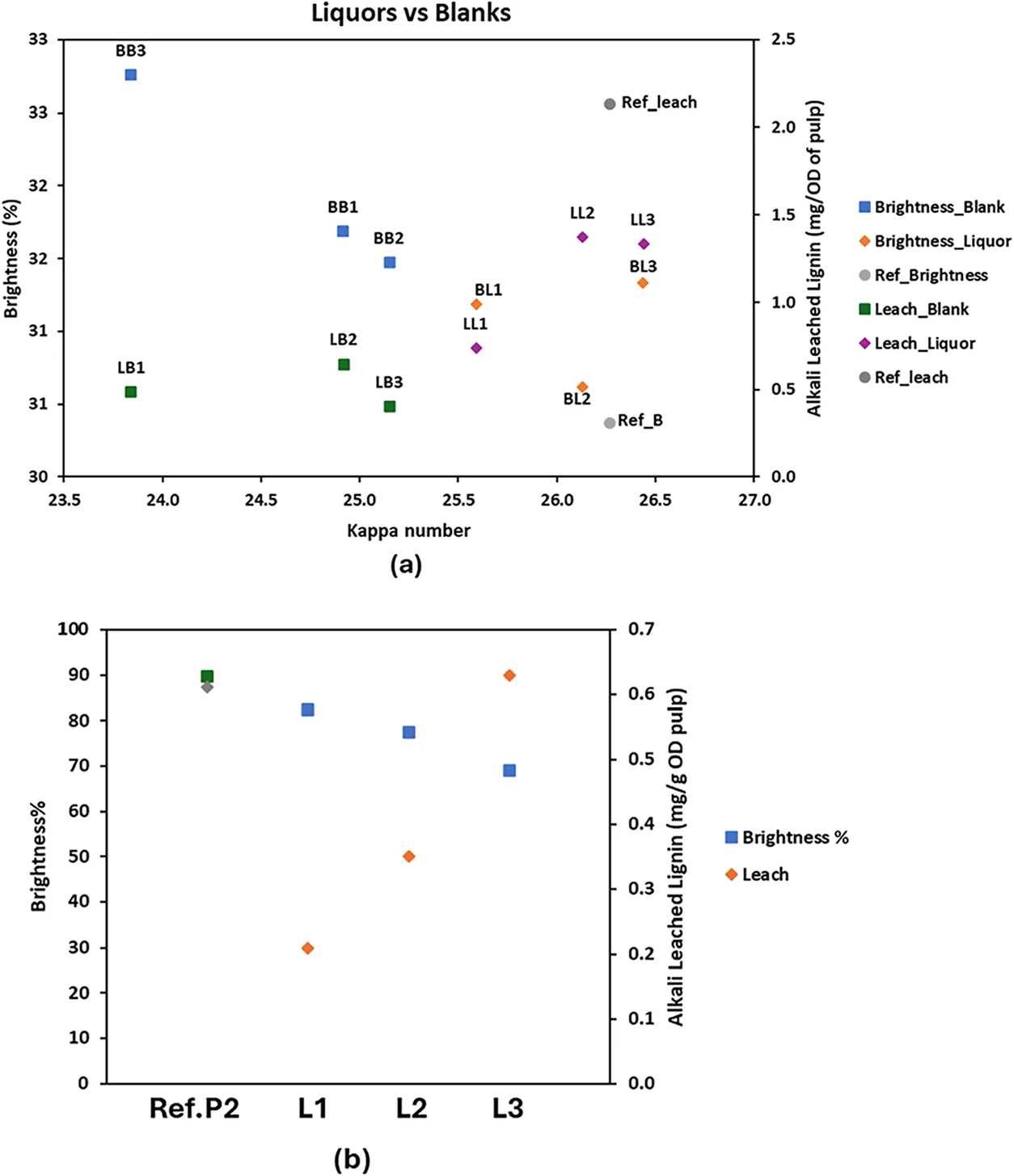
Effects of liquor composition on lignin redeposition and removal efficiency for (a) unbleached pulp (P1) and (b) bleached pulp (P2). Kappa number, brightness, and alkali-leached lignin are shown as indicators of lignin retention. Ref_B—Brightness reference, Ref_leach—Leach reference. BB—brightness blank, LB—blank leach, LL—leach liquor, BL—blank liquor. Industrial liquors (L1–L3) promoted greater redeposition than their corresponding blank solutions (B1–B3), reflecting the influence of lignin and ion content.

**Table 5:** Properties of Unbleached Pulp (P1) after Treatment with Industrial Liquors (L1, L2, and L3) and Corresponding Blanks (B1, B2, B3)

unbleached	Kappa number	brightness %	alkali-leached lignin (mg/g OD Pulp)
L1	25.6	31.2 ± 0.8	0.7
L2	26.1	30.6 ± 0.5	1.4
L3	26.4	31.3 ± 0.5	1.3
B1	24.9	31.7 ± 0.5	0.6
B2	25.1	31.4 ± 0.3	0.4
B3	23.8	32.7 ± 0.9	0.5

The greater lignin retention observed in the higher-lignin liquors may be partly explained by mass-transfer limitations. As the lignin concentration in the surrounding liquor increases, the concentration gradient driving the diffusion of dissolved lignin from the fiber wall decreases, which may reduce the net rate of lignin removal.^25^

Notably, no consistent correlation was observed between kappa number, brightness, and leached lignin for L1–L3. This may partly reflect differences in the properties of the lignin present in the different liquors, particularly in L3; however, molecular weight and structural analysis would be required to confirm this hypothesis. For bleached pulps (Figure 2b), the trends observed for L1, L2 and L3 were generally similar to those observed for unbleached pulps (Figure 2a), although the magnitude of the changes was different. This indicates that liquor composition had a significant influence on lignin retention and redeposition behavior in both pulps.

Overall, treatment with the higher-strength industrial liquors was associated with greater lignin retention, as indicated by lower brightness and higher kappa numbers compared with the corresponding blanks. The blank treatments generally resulted in higher brightness and lower kappa numbers, although the differences in brightness between blank- and liquor-treated pulps were relatively small (<1% in some cases). Together, these results indicate that the components present in the industrial liquors influence the balance between lignin removal and retention during washing. The higher lignin concentrations and conductivity of these liquors may contribute to increased lignin retention through a combination of reduced mass-transfer driving force and enhanced lignin association or redeposition.

### 4.3 Effects of pH at Constant Composition on Liquor Removal and Reattachment

The influence of pH was examined using liquor L3, which had the highest lignin concentration and conductivity among the investigated liquors (Table 4). Lowering the pH by H_2_SO_4_ addition increased lignin retention in the pulp, as indicated by higher kappa numbers, lower brightness, and greater amounts of leached lignin (Figure 3). Conversely, increasing the pH by NaOH addition reduced lignin redeposition, with the kappa number decreasing by up to 0.9 units (Figure 3a). However, the effect of pH adjustment on brightness and leached lignin were relatively small. Between the lowest and highest pH conditions, the difference in brightness was only about 2% (Figure 3a), while the difference in leached lignin was approximately ∼1 mg/g (Figure 3b). Within the alkaline pH range investigated here, decreasing pH increased lignin retention. This is consistent with a reduction in lignin ionization and electrostatic stabilization as alkalinity decreases, which can reduce lignin solubility and favor association or deposition. However, because the investigated pH values remained above approximately 12, this effect should not be interpreted simply as precipitation caused by lowering the pH below the phenolic p*K*_a_. Changes in residual alkalinity, ionic strength and liquor composition may also contribute.

**Figure 3:**
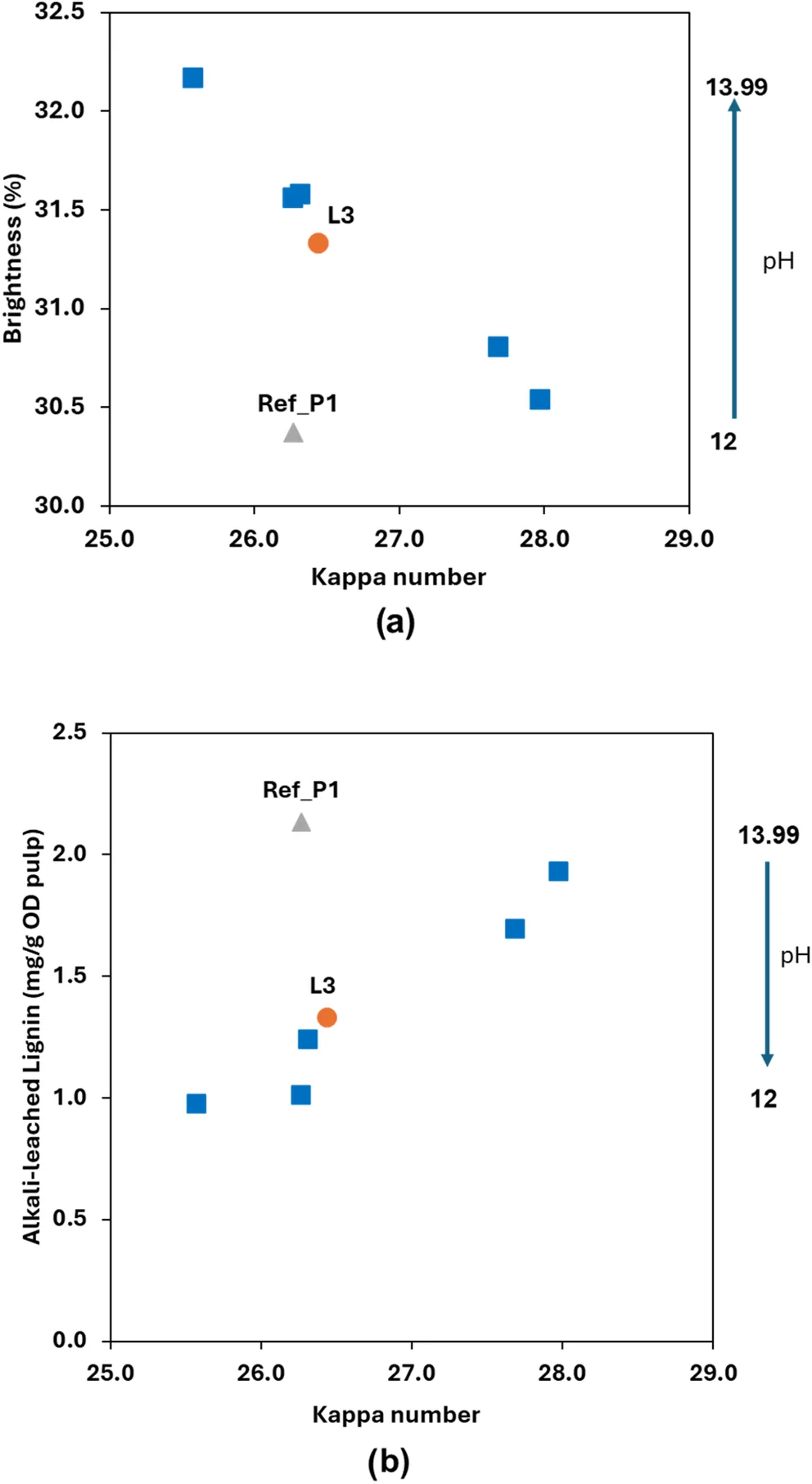
Effect of pH adjustment in liquor (L3) on unbleached pulp. Brightness (a) and alkali-leached lignin (b) as functions of kappa number.

### 4.4 Combined Influence of Liquor Composition and pH

To distinguish the effect of liquor compositions from those of pH alone, liquors B1, L1, and L3 were adjusted to the same pH as L1 (12.45). Despite the identical pH, lignin retention generally increased with increasing lignin concentration and conductivity of the liquors (Figure 4). Pulp treated with L3, adjusted to pH 12.45, exhibited a higher kappa number, lower brightness (Figure 4a) and a greater amount of leached lignin than pulp treated with L1 or the blank solution B1 (Figure 4b). The pH-adjusted B1 and L1 samples are included in Figure 4 together with the adjusted L3 sample for comparison. These findings indicate that liquor composition significantly influenced lignin retention, even when pH was held constant. The observed differences may reflect the combined effect of lignin concentration, ionic composition, and other liquor characteristics. Possible differences in lignin molecular weight distribution and chemical structure may also contribute; however, these properties were not characterized in the present study.

**Figure 4:**
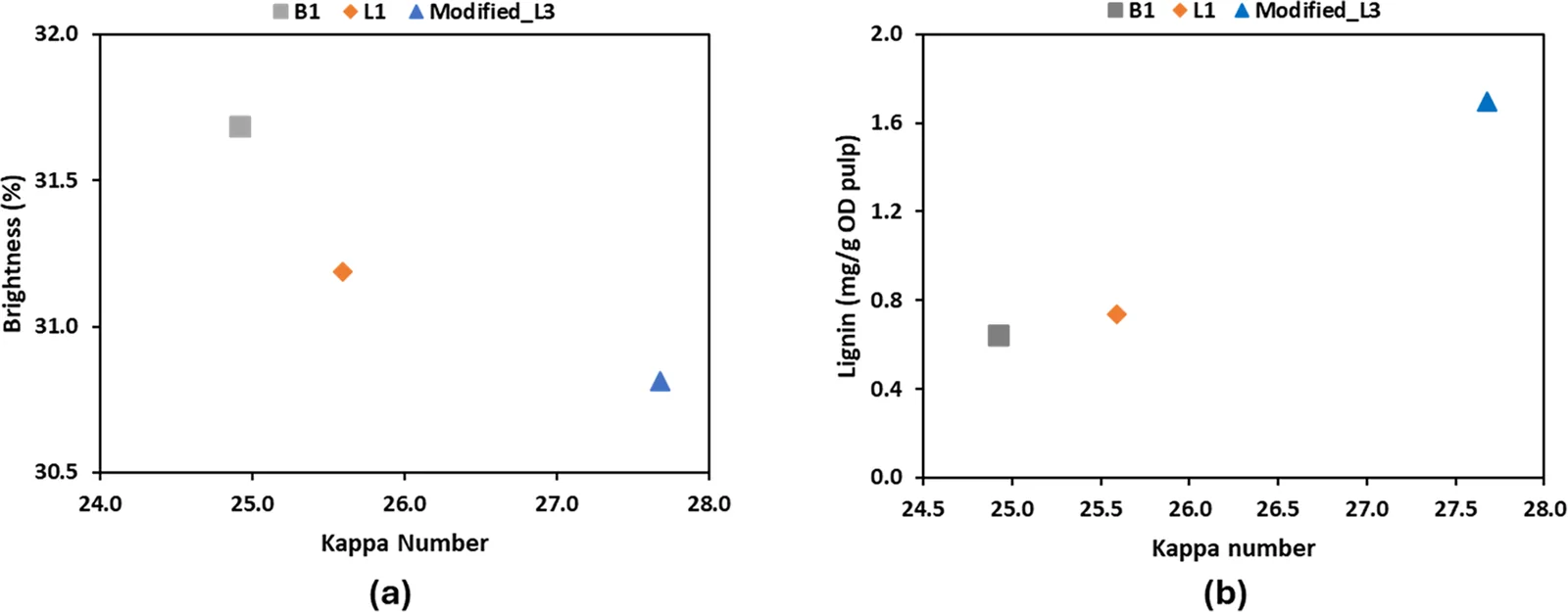
Effects of liquor composition at constant pH (12.45) on unbleached pulp. Brightness (a) and alkali-leached lignin (b) as functions of kappa number.

### 4.5 Effects of Alkali Addition (NaOH)

The influence of NaOH addition was evaluated by comparing liquor L3 (highest lignin and ion content) with L2 (lower lignin contentand residual alkali than L3). For unbleached pulp (P1), increasing NaOH concentration improved lignin removal in both liquors; however, the response differed. L2 showed a strong effect at 2 g/L NaOH, whereas L3 required ≥ 5 g/L to achieve a comparable reduction in redeposition. In both cases, a threshold effect was observed: further alkali addition beyond this point did not significantly enhance removal. Brightness (Figure 5a,5b) improved more in L2 (up to +1.5%) than in L3 (+0.8%), while leached lignin (Figure 5c,5d) decreased slightly more in L3 (−0.5 mg/g) than in L2 (−0.4 mg/g). The maximum reduction in kappa number was 0.9 units for L3 and 0.5 units for L2.

**Figure 5:**
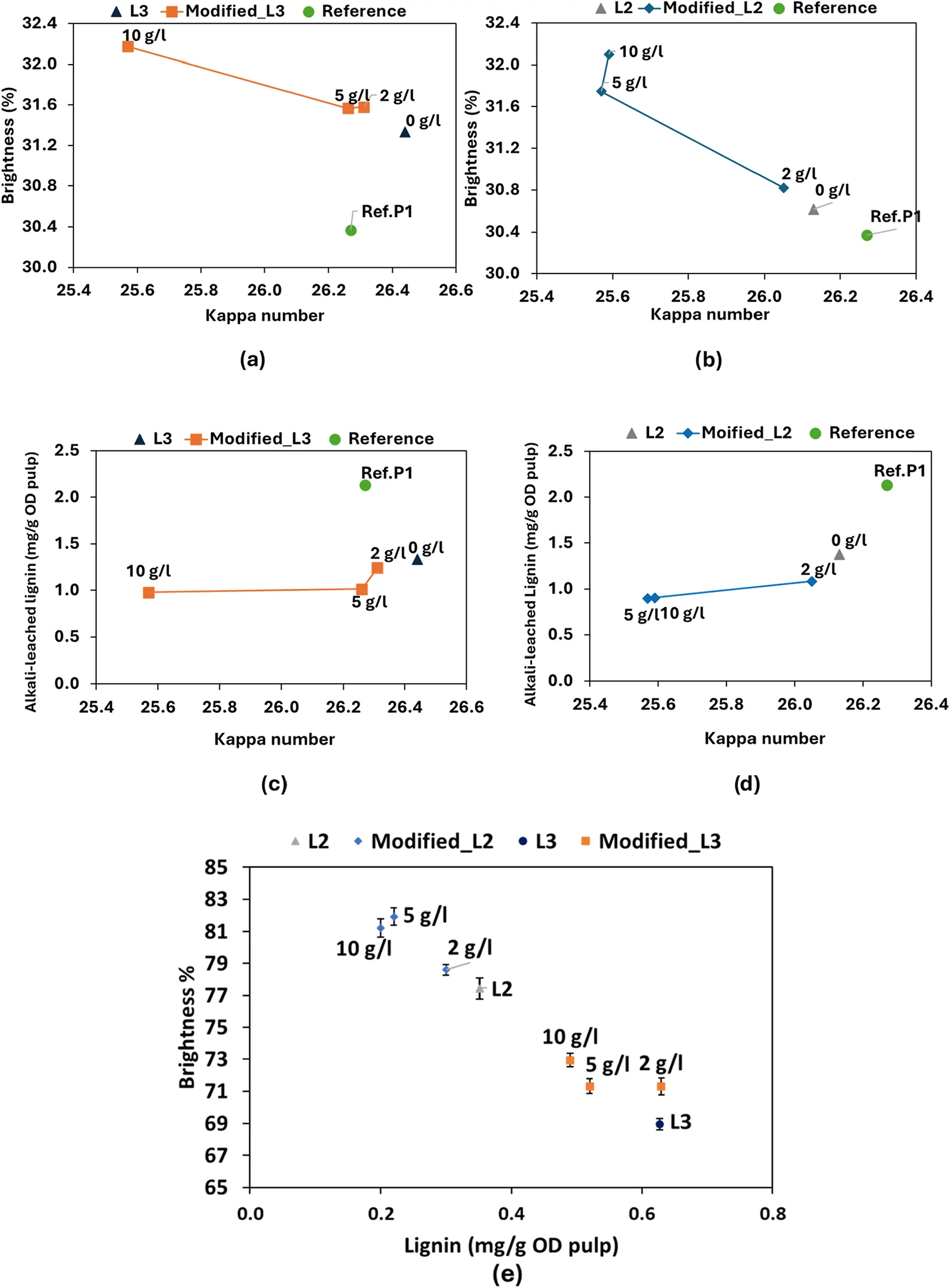
Effect of NaOH addition (2, 5, and 10 g/L) on lignin redeposition and removal in unbleached pulp (P1) and bleached pulp (P2): (a, b) brightness versus kappa number for P1 treated with L3 and L2, respectively; (c, d) alkali-leached lignin content versus kappa number for P1 treated with L3 and L2, respectively; and (e) brightness versus lignin content for P2 treated with L2 and L3. Modified_L2 and Modified_L3 denote liquors supplemented with NaOH; 0 g/L denotes treatment without added NaOH. ref P1 denotes the reference unbleached pulp. Lignin contents are expressed per gram of oven-dry (OD) pulp.

For bleached pulp (P2), the overall response to NaOH addition was weaker. At the highest NaOH concentration, both liquors showed nearly identical effects: brightness increased by ∼4% and leached lignin decreased by ∼0.15 mg/g (Figure 5e). These results suggest that the addition of NaOH primarily benefits liquors with low residual alkali, and that its effect levels off once lignin solubility is restored. From an industrial standpoint, adding excess NaOH is expensive and provides little benefit, emphasizing the importance of optimizing alkali dosage for efficient washing.

### 4.6 Effects of Divalent Cations (Mg^2+^ from MgSO_4_)

The effect of magnesium ions was assessed by adding MgSO_4_ to liquor L2 to simulate possible carryover from oxygen delignification. At mill-relevant concentrations of ∼1 g/L, MgSO_4_ showed little or no change in lignin retention, consistent with earlier findings by Sundin (2000). One possible explanation is that the ions already present in L2 influence the fiber sorption sites and solution chemistry, such that a small additional Mg^2+^ input has only a limited effect on the overall ionic environment.^17,20^ In contrast, the deliberately elevated MgSO_4_ concentration of 10 g/L resulted in substantially greater lignin retention, as indicated by an increase in kappa number of up to 1.5 units, decreased brightness (Figure 6a), and increased amounts of leached lignin (Figure 6b). The intermediate concentrations of 3 g/L produced more variable results, indicating a concentration-dependent response that may also be influenced by interactions with other liquor components. The observed behavior is consistent with previously proposed effects of multivalent cations on lignin colloidal stability, including charge screening, aggregation, and possible ion bridging.^3,23^ In addition, Mg^2+^ can sorb onto fiber surfaces through ion exchange at carboxyl groups in hemicelluloses and residual lignin,^15,19^ potentially altering fiber surface charge and lignin–fiber interactions. However, these mechanisms were not directly investigated in the present study.

**Figure 6:**
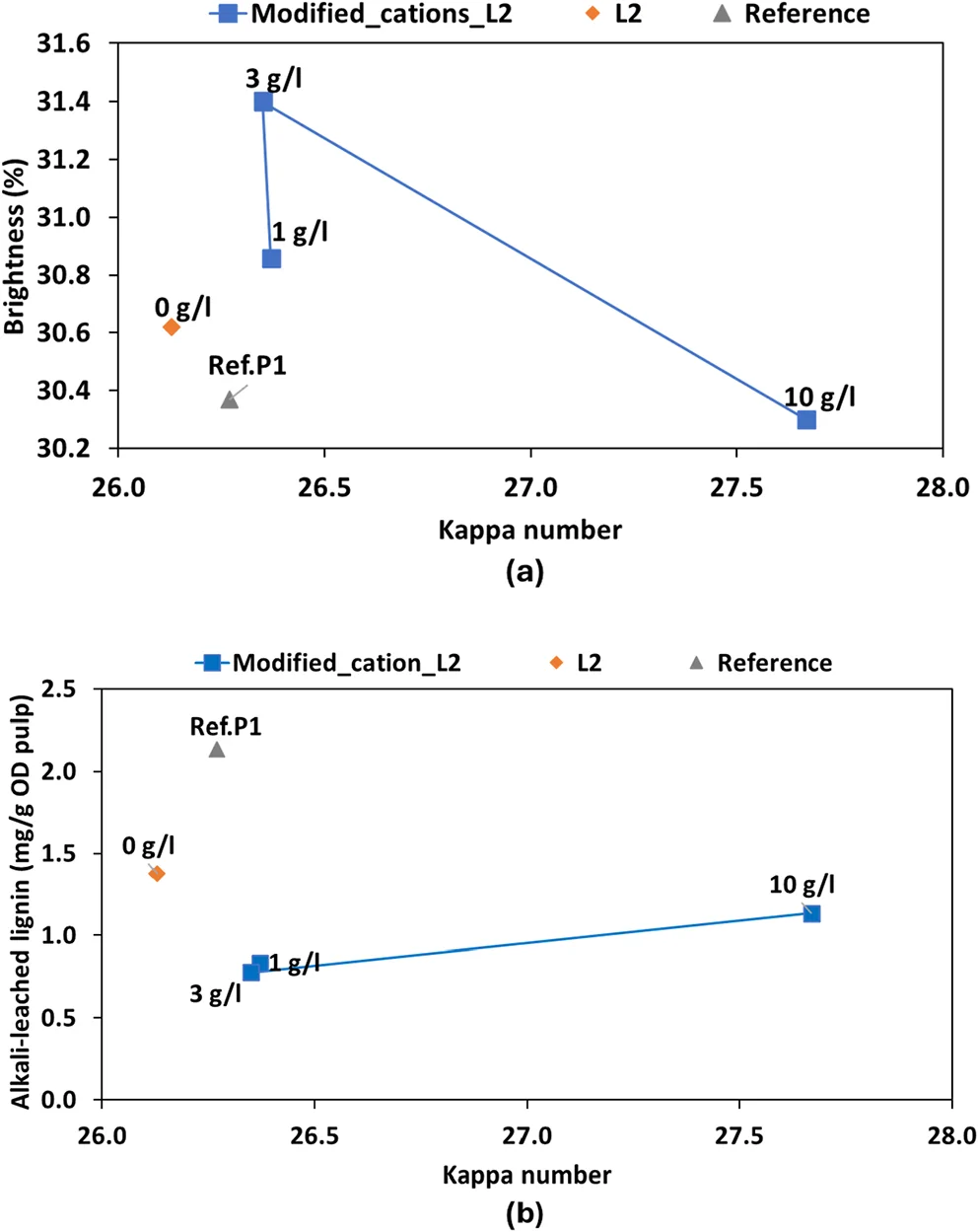
Effect of MgSO_4_ addition (1, 3, and 10 g/L) to liquor L2 on lignin redeposition in unbleached pulp (P1). Brightness (a) and alkali-leached lignin (b) as functions of kappa number.

## 5 Discussion

### 5.1 Mechanistic Interpretation of Lignin Redeposition

Lignin redeposition reflects a dynamic balance between lignin removal and reattachment, influenced by liquor composition, pulp type, and washing conditions. In unbleached pulp with a high initial lignin content, lignin removal and retention may occur simultaneously, complicating the interpretation of washing behavior. As illustrated in Figure 7, parameters such as pH, temperature, and multivalent cations (Ca^2+^ and Mg^2+^) can influence lignin solubility, colloidal stability, and aggregation. Decreasing pH reduces the degree of lignin ionization and may therefore favor lignin association and precipitation, while divalent cations can screen or partially neutralize negative charges and promote aggregation.

**Figure 7:**
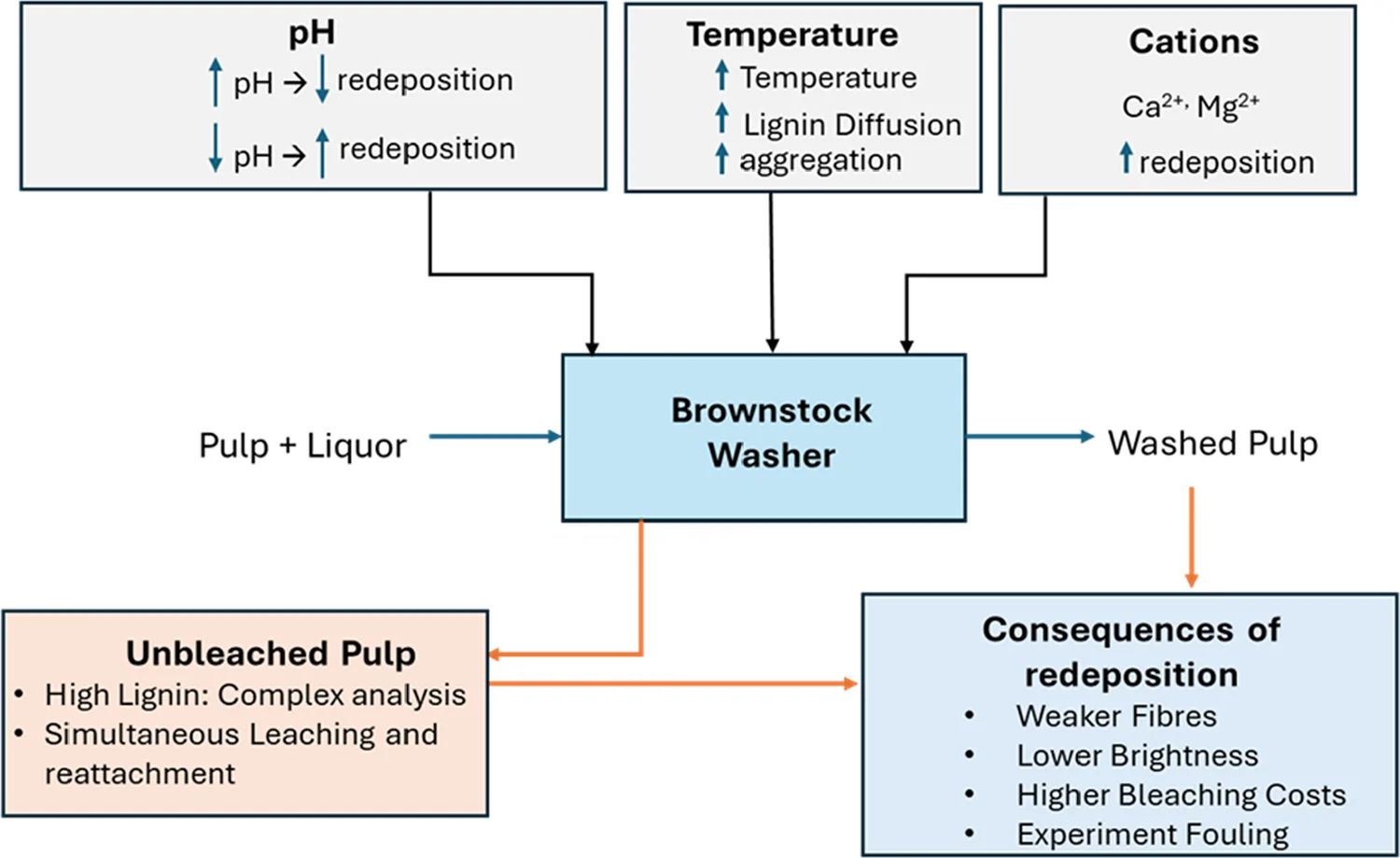
Factors influencing lignin redeposition during brownstock washing.

Divalent cations can interact not only with dissolved lignin but also with pulp fibers through competition with monovalent cations (Na^+^ and K^+^) for binding sites, particularly carboxyl groups in hemicelluloses and residual lignin.^15,16,18,19^ Binding of Mg^2+^ or Ca^2+^ to these sites can alter the fiber surface charge and thereby modify the electrostatic interactions between fibers and negatively charged lignin fragments, potentially influencing lignin adsorption.^17,20^ These effects may also be influenced by other salts present in the liquor, such as sodium sulfate, which has been reported to affect hydrophobic interactions and lignin reattachment.^21^ However, the sodium sulfate concentration was not measured in the present liquors; therefore, its contribution cannot be evaluated. Together, these mechanisms can contribute to increased lignin retention on fibers, reduced pulp brightness, and potentially higher downstream bleaching demand.

Our findings are consistent with those of Maximova et al.,^7^ who proposed that precipitation is an important mechanism of lignin deposition and that weakly bound lignin can be removed during washing, whereas more strongly bound fractions may remain attached to the fibers. The extent of washing can therefore influence the observed amount of retained lignin and complicate comparisons between studies. Furthermore, differences in raw material, lignin properties, liquor composition, and washing protocols may influence which lignin fractions are removed or retained, contributing to variability in experimental results.

### 5.2 Scope, Evidence, and Limitations

The pH response observed in the present study should not be interpreted as a universal quantitative relationship between pH and lignin redeposition. Industrial liquors are complex multicomponent systems, in which lignin concentration, residual alkali, ionic strength, and dissolved lignin characteristics may vary simultaneously. Within the investigated alkaline pH range, decreasing pH was generally associated with increased lignin retention. However, comparisons at constant pH showed that the liquor composition also had a significant influence on lignin retention. Establishing a predictive relationship between pH and lignin redeposition would require a more extensive data set in which pH, ionic strength, cation type, lignin concentration, and temperature are varied independently.

The combined measurements of kappa-number, brightness, UV–vis, and alkaline leaching offer consistent evidence for changes in lignin retention. However, they do not clarify the molecular structure or the molar mass distribution of the redeposited fraction. Techniques such as SEC/GPC, FTIR, and NMR, along with UV–vis spectral analysis, could help determine whether specific lignin fractions or functional groups tend to aggregate or bind to fibers. Recent research on lignin self-assembly highlights that molecular weight, phenolic groups, hydrophobic interactions, and electrostatic screening collectively influence aggregation.^28^ Such characterization methods, combined with zeta-potential or particle-size measurements, are valuable for more directly understanding the Mg^2+^ mechanism. This approach is beyond the scope of the current study, which primarily relies on pulp-level evidence obtained under controlled washing conditions.

Overall, the schematic (Figure 7) emphasizes how operating conditions in the brownstock washer directly control the extent of lignin redeposition and its downstream consequences for pulp quality and mill performance.

### 5.3 Industrial Implications

Our results highlight several practical considerations. In closed-mill systems, nonprocess elements (NPEs), including calcium and magnesium, can accumulate during the recovery cycle.^4,5,29^ Brownstock filtrate is returned with weak black liquor to evaporation and recovery; consequently, Mg^2+^ entering this stream is not only a fibreline concern. Magnesium is primarily associated with inorganic solids and purge streams, such as green liquor dregs and lime mud. Changes in Mg^2+^ loading can alter dregs composition, settling, and cake-filtration behavior, making green-liquor clarification and dregs handling relevant when Mg^2+^ carryover or makeup increases.^29–31^ The direction and magnitude of the effect depend on the overall NPE inventory and solids chemistry; therefore, the present pulp-scale experiments cannot predict mill filterability. A mill-specific Mg^2+^ balance and monitoring of green-liquor dregs should therefore be considered before translating the laboratory findings into operating changes.

NaOH addition also has recovery-cycle and economic consequences. Alkali added before or during brownstock washing increases the amount of sodium entering the weak-black-liquor circuit upon filtrate recovery. Unless compensated by lower sodium makeup elsewhere, this changes the mill sodium inventory and may shift the sodium-to-sulfur balance and green liquor sulfidity. Based on the experimental treatment ratio of 138 mL liquor mixture per 20 g oven-dry pulp, equivalent to 6.9 m^3^/ADT, NaOH concentrations of 2, 5, and 10 g/L correspond to nominal laboratory charges of 13.8, 34.5, and 69.0 kg NaOH/ADT, respectively. These should not be interpreted as recommended full-scale charges. Moreover, purchased NaOH adds to direct operating costs, while additional dissolved sodium contributes to the inorganic load handled during evaporation and recovery. Because the improvement plateaued, routine overaddition would be difficult to justify. The practical implication is to preserve and control residual alkalinity and to optimize the location and dosage of existing alkali inputs, supported by a mill sodium–sulfur balance and an economic assessment, rather than indiscriminately applying NaOH as a new washing chemical.

Finally, conventional brownstock-washer performance is typically evaluated using measures such as COD, dissolved solids carryover, wash loss, displacement ratio, and dilution factor. These were not determined in this work. Kappa number, brightness, UV–vis lignin content, and alkaline-leached lignin instead describe a specific component of washing behavior: the removal and reattachment of lignin. Accordingly, the results identify chemical conditions that may influence pulp cleanliness and downstream bleaching demand, but they do not demonstrate an overall change in industrial washer efficiency.

Altogether, the results support monitoring liquor pH, residual alkalinity, and Mg^2+^ carryover as part of an integrated brownstock-washing strategy. Translation to mill operation must also account for conventional carryover metrics, the sodium–sulfur balance, recovery-cycle NPE purges, green-liquor clarification, chemical cost, and the point at which further alkali addition no longer improves lignin removal.

## 6 Conclusions

Lignin redeposition during brownstock washing was strongly influenced by liquor composition and the chemical conditions investigated in this study. Within the studied alkaline pH range, decreasing pH was generally associated with increased lignin retention on fibers, as indicated by higher kappa numbers and lower brightness. NaOH addition reduced lignin redeposition, but the response plateaued beyond a liquor-dependent threshold. The mill-relevant MgSO_4_ condition of 1 g/L produced little change, whereas the deliberately elevated 10 g/L condition increased lignin retention. This concentration-dependent response is consistent with previously proposed effects of Mg^2+^ on lignin colloidal stability and aggregation, although the underlying mechanism was not directly investigated in the present study. Compared with bleached pulp, unbleached pulp showed a more pronounced interplay between lignin removal and retention, consistent with its higher initial lignin content. These findings specifically concern lignin removal and retention rather than overall washer efficiency, which requires conventional measurements such as COD, dissolved solids carryover, wash loss, displacement ratio and dilution factors. The results therefore support monitoring and controlling liquor chemistry, including pH, residual alkalinity, and Mg^2+^ carryover, while any translation to mill operation should also consider sodium–sulfur balance, recovery-cycle NPE management, green-liquor filtration, and chemical cost.
